# Computationally modeling interpersonal trust

**DOI:** 10.3389/fpsyg.2013.00893

**Published:** 2013-12-04

**Authors:** Jin Joo Lee, W. Bradley Knox, Jolie B. Wormwood, Cynthia Breazeal, David DeSteno

**Affiliations:** ^1^Media Lab, Massachusetts Institute of TechnologyCambridge, MA, USA; ^2^Department of Psychology, Northeastern UniversityBoston, MA, USA

**Keywords:** computational trust model, interpersonal trust, machine learning, nonverbal behavior, social signal processing, human-robot interaction

## Abstract

We present a computational model capable of predicting—above human accuracy—the degree of trust a person has toward their novel partner by observing the trust-related nonverbal cues expressed in their social interaction. We summarize our prior work, in which we identify nonverbal cues that signal untrustworthy behavior and also demonstrate the human mind's readiness to interpret those cues to assess the trustworthiness of a social robot. We demonstrate that domain knowledge gained from our prior work using human-subjects experiments, when incorporated into the feature engineering process, permits a computational model to outperform both human predictions and a baseline model built in naiveté of this domain knowledge. We then present the construction of hidden Markov models to investigate temporal relationships among the trust-related nonverbal cues. By interpreting the resulting learned structure, we observe that models built to emulate different levels of trust exhibit different sequences of nonverbal cues. From this observation, we derived sequence-based temporal features that further improve the accuracy of our computational model. Our multi-step research process presented in this paper combines the strength of experimental manipulation and machine learning to not only design a computational trust model but also to further our understanding of the dynamics of interpersonal trust.

## 1. Introduction

Robots have an immense potential to help people in domains such as education, healthcare, manufacturing, and disaster response. For instance, researchers have designed robots that take steps toward helping children learn a second language (Kanda et al., 2004), assisting nurses with triage (Wilkes et al., 2010), and participating as part of a search and rescue team (Jung et al., 2013). As such robots begin to collaborate with us, we should consider mediating interpersonal or social factors that can affect the outcome of the human-robot team. Methods for incorporating pro-social interpersonal factors like trust, friendliness, engagement, rapport, and comfort, when designed in a way that is appropriate across different contexts, can enable socially assistive robots to develop cooperative relations with their human partners. Trust, in particular, has been shown to facilitate more open communication and information sharing between people (Maddux et al., 2007). Thus by establishing an appropriate sense of trust, robots may become more effective communicators and thereby increase their capacity to function as collaborative partners. When designing for such interactions, we need to answer how a robot can (1) *behave* such that humans develop trust toward the robot (i.e., the control signal). But to evaluate the effectiveness of such behavior, we first ask how a robot can (2) *evaluate* the degree to which an individual trusts the robot (i.e., feedback signal). Considering the development of trust from the perspective of control systems, a robot can continuously adapt its behavior to achieve a desired level of trust through *a feedback signal that assesses a person's current level of trust toward the robot*.

This paper focuses on the development of a system that can infer the degree of trust a human has toward another social agent. For the purposes of the current investigation, we utilize a behavioral operationalization of trust as a willingness to cooperate for mutual gain even at a cost to individual asymmetric gain and even when such behavior leaves one vulnerable to asymmetric losses. In this paper, *trusting behavior* represents a person's willingness to cooperate with his partner and *trustworthiness* represents his partner's willingness to cooperate, which the person assesses before potentially engaging in trusting behavior.

We present a computational model capable of predicting—above human accuracy—the subsequent trusting or distrusting behavior of an individual toward a novel partner, where these predictions are based on the nonverbal behaviors expressed during their social interaction. We predict trusting behaviors using a machine learning approach. Specifically, we employed supervised learning methods, which present a broad toolset that focuses on accurate prediction of the values of one or more output variables given the values of an input vector. There is certainly much overlap in supervised learning and statistical methods in psychology, with many of the same techniques going by different names, but supervised learning places more emphasis on the accuracy of the learned/fit model and permits a wider range of modeling techniques, both probabilistic/statistical and otherwise.

Our work consists of three major parts and is organized into the following phases:

- **Trust-Related Nonverbal Cues (Phase 1)**: We include a summary of our prior work, in which we identify nonverbal cues that, when expressed by a social entity (i.e., humans or expressive robots), are perceived as signals of untrustworthy behavior.
- **Design of Prediction Model (Phase 2)**: We use the results from Phase 1 to inform the design of a computational model capable of predicting trust-related outcomes. We compare its prediction performance to a baseline model that is not informed by our Phase 1 results, a random model, an *a priori* model, and the predictions of human participants.
- **Temporal Dynamics of Trust (Phase 3)**: To improve the accuracy of the prediction model developed in Phase 2, we derive additional features that capture the temporal relationships between the trust-related cues. With the addition of these features, our computational model achieves significantly better performance than all the baseline models as well as human judgement.

## 2. Background

In situations where a person's past behaviors or reputation are unknown, we rely on other possible sources of information to infer a person's intentions and motivations. Nonverbal behaviors are a source of information about such underlying intentions, goals, and values and have often been explored as “honest” or “leaky” signals. These signals are primitive social signals that are thought to occur largely outside of people's conscious control (Pentland, 2008; Ambady and Weisbuch, 2010; Knapp and Hall, 2010). Nonverbal behaviors include body language, social touch, facial expressions, eye-gaze patterns, proxemics (i.e., interpersonal distancing), and vocal acoustics such as prosody and tone. Through these nonverbal expressions, we communicate mental states such as thoughts and feelings (Ambady and Weisbuch, 2010).

Researchers working in domains such as human communication modeling and social signal processing have worked toward modeling and interpreting the meaning behind nonverbal behavioral cues (Morency, 2010; Vinciarelli et al., 2012). By observing the nonverbal communication in social interactions, researchers have predicted outcomes of interviews (Pentland, 2008) and the success of negotiations (Maddux et al., 2007). In other work, by observing head, body, and hand gestures along with auditory cues in speech, a hidden conditional random field model could differentiate whether a person was agreeing in a debate with accuracy above chance (Bousmalis et al., 2011). In similar work, Kaliouby and Robinson (2004) used a dynamic Bayesian network model to infer a person's mental state of agreement, disagreement, concentration, interest, or confusion by observing only facial expressions and head movements. Other research has tried to model cognitive states, like frustration (Kapoor et al., 2007), and social relations like influence and dominance among groups of people (Jayagopi et al., 2009; Pan et al., 2011). However, this article describes the first work toward computationally predicting the trusting behavior of an individual toward a social partner.

To the best of our knowledge, trust-recognition systems currently exist only in the context of assessing trust and reputation information among buyers and sellers in online communities. By observing transaction histories, consumer ratings of sellers, and peer-to-peer recommendations, online services like Amazon and eBay utilize these computational models to decide whether an online service or product is trustworthy or reputable in the electronic marketplace (Pinyol and Sabater-Mir, 2013).

Research on detecting deception has also taken a behavioral approach to computational modeling, focusing on the atypical nonverbal behaviors produced by the cognitive effort of concealing the truth (Meservy et al., 2005; Raiman et al., 2011). Although related to the concept of trust, research on deception focuses narrowly on detecting purposeful deception and distinguishing lies from truths, whereas this article focuses more broadly on understanding how much an individual trusts another person in more natural social encounters.

We suspect that the absence of work on predicting trust in face-to-face social interaction comes in part from the uncertainty about which nonverbal behaviors contain predictive information for trust-related outcomes. Thus, we began our investigation with a search for social signals that help predict the trustworthiness of an unfamiliar person in a social interaction.

## 3. Trust-related social signals (phase 1)

In this section, we summarize the key findings of our prior work (for further details see DeSteno et al., 2012), in which we identified a set of nonverbal cues that is indicative of untrustworthy behavior. We also demonstrated people's readiness to interpret those same cues to infer the trustworthiness of a social humanoid robot.

After meeting someone for the time first, we often have a sense of how much we can trust this new person. In our prior work, we observed that when individuals have access to the nonverbal behaviors of their partner in a face-to-face conversation, they are more accurate in predicting their partner's trust-related behavior than when they only have access to verbal information in a web-based chat.

Building from this result, we then investigated which specific nonverbal cues signal subsequent trusting or distrusting behavior. We hypothesized that the appearance of multiple nonverbal cues together, observed in the context of each other, would provide such predictive information as opposed to a single “golden cue” offering predictive ability. Rather than assuming a one-to-one correspondence between a specific behavior and its underlying meaning, we viewed the interpretation of nonverbal cues as highly context dependent. That is, by observing multiple cues in close temporal proximity, we gain more meaningful interpretations than by independently assessing the meaning behind a single cue. For example, an eye-roll in conjunction with a large grin can be more accurately interpreted as conveying humor, whereas observing an eye-roll in isolation could lead to the less accurate interpretation of contempt. The interpretation of the eye-roll is contingent upon the observation of the grin. We operationalized this contextual dependency through a mean value of occurrences across a set of nonverbal cues seen within an interaction (i.e., the mean frequency of cues).

We identified four nonverbal cues—face touching, arms crossed, leaning backward, and hand touching—that as a set are predictive of lower levels of trust. We found that increased frequency in the joint expression of these cues was directly associated with less trusting behavior, whereas none of these cues offered significant predictive ability when examined in isolation. In our experiments, trusting behavior was measured as participants' exchange action with their partner during an economic game (the game details are in section 4.1.3). Thus, the more frequently an individual *expressed* these cues, the less trusting was their behavior toward their partner in this game.

**Figure 1:**
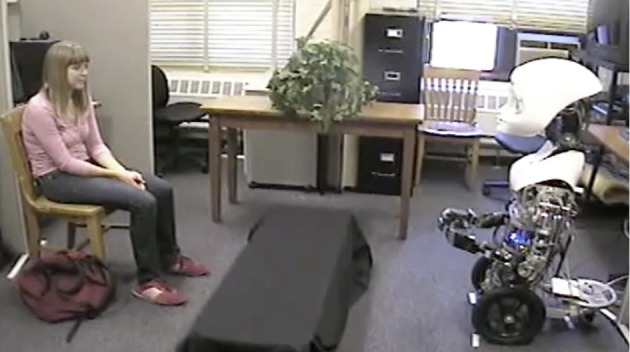
**A participant engaging in a 10-min conversation with a teleoperated humanoid robot, Nexi, here expressing the low-trust cue of hand touching**.

To confirm these findings, we validated the nonverbal cue set through a human-subjects experiment in which we manipulated the occurrence of nonverbal cues exhibited by a humanoid robot. By utilizing a social robotic platform, we took advantage of its programmable behavior to control exactly which cues were emitted to each participant. Participants engaged in a social conversation with a robot that either (a) expressed neutral conversational gestures throughout the interaction, or (b) replaced some of those gestures with each of the four target cues (shown in Figure 1). As predicted, the robot's expression of the target cues resulted in participants perceiving the robot as a less trustworthy partner, both in terms of participants' self reports as well as their trusting behavior (i.e., game exchange behavior) toward the robot. Thus, when individuals *observed* these cues from the robot, they trusted their robot partner less.

From these human-subjects experiments conducted as part of our prior work (DeSteno et al., 2012), we extract three key findings that serve as guidelines to help inform the design of our computational trust model.

1. There exists a set of four nonverbal cues associated with lower levels of trust: face touching, arms crossed, leaning backward, and hand touching.
2. The joint appearance of cues, represented by their mean frequency, results in a stronger signal that is predictive of trusting outcomes, whereas the cues individually possess limited predictive power.
3. These nonverbal cues predict the trust-related behaviors of an individual that is either expressing or observing them.

## 4. Design of prediction model (phase 2)

In this section, we incorporate the guidelines gained from our human-subjects experiments in Phase 1 into the design of a computational model capable of predicting the degree of trust a person has toward a novel partner, which we will refer to as the “trust model” for brevity. By utilizing this domain knowledge in the feature engineering process, we are able to design a prediction model that outperforms not only a baseline model built in naiveté of our prior findings but also outperforms human accuracy.

### 4.1. Materials (phase 2)

First we describe the data collection material consisting of the human-subjects experiment, the operationalization of trust through an economic exchange game, and the video-coded annotations of the participants' nonverbal behavior. This data corpus is used to train and evaluate our trust model. We then describe our methods for model design, consisting of our strategies for feature engineering, the nested cross-validation method to estimate the model's true error, and the learning algorithm and model representation selected to create our prediction model.

#### 4.1.1. Data collection material

We leverage the pre-existing datasets from our human-subjects experiments in which the task scenario involved two participants interacting for 5-min and then asked to make trust judgements of one another. A total of 56 interaction pairs or 112 people participated in these studies. Some of this data (20 interaction pairs) originated from the human-human study described in Phase 1, and the remaining are from a separate study. The pool of participants was undergraduates attending Northeastern University in Boston, Massachusetts. 31% of the participants were male and 69% were female. The data collection materials included the raw videos of the human-subjects experiments, video-coded annotations of the participants' nonverbal behaviors, and trust measurements obtained through the Give-Some Game described in section 4.1.3.

#### 4.1.2. Human-subjects experiment

**Figure 2:**
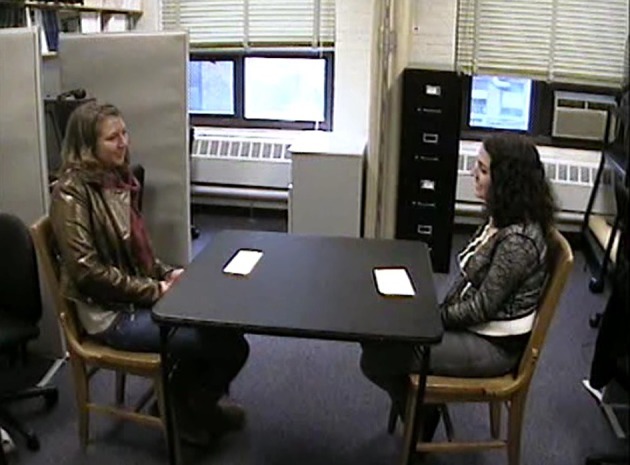
**Lab room setup for human-subjects experiment.** Participants engaged in a “get-to-know-you” interaction with another random participant. Slips of paper on the table listed some conversation topic suggestions.

The experiments consisted of two parts. Participants first engaged in a 5-min “get-to-know-you” interaction with another random participant (whom they did not know prior to the experiment). This part of the study was held in a quiet room, where participants were seated at a table as shown in Figure 2. The participants were encouraged to discuss anything other than the experiment itself. To facilitate conversation, topic suggestions such as “Where are you from?” and “What do you like about the city?” were placed upon the table on slips of paper. Around the room, three time-synced cameras captured the frontal-view of each participant along with a side-view of the participants (the perspective shown in Figure 2). For the second half of the experiment, the interaction partners played the Give-Some Game, explained below. The participants were not told that they would play this cooperative game with their conversational partner until after the “get-to-know-you” period was over.

#### 4.1.3. Operationalization of trust

A participant's judgement of trust toward their novel partner was behaviorally measured through the Give-Some Game (Lange and Kuhlman, 1994). The Give-Some Game is similar to a traditional Prisoner's Dilemma game in that it represents a choice between self-interested behavior and cooperative behavior (DeSteno et al., 2010). At the game's start, each player possesses four tokens. Each token is worth $1 to the player and $2 in the possession of their partner. Each player decides how many of the four tokens to give to their partner, keeping the remaining tokens for themself. For maximum individual payoff, a player must keep all four tokens. This strategy ensures that the player receives at least $4 (while giving nothing to their partner); anything they receive from their partner would further increase their payoff. For maximum communal benefit, both of the players would need to give away all four tokens to the other, resulting in each player earning $8.

The participants are separated into different rooms to prevent the communication of strategies. To limit apprehension about having to face a partner to whom they were selfish, participants are also told that they will not see each other again. Although the game is played individually, the outcome (the money a player wins) depends on the decisions made by both the players in the game. In the game, players are asked to:

1. Decide how many tokens they want to give to their partner.
2. Predict how many tokens they believe their partner will offer them.

In this article, we consider the number of tokens a participant gives to represent how much they trust their partner to play cooperatively. In addition, we consider the discrepancy between the predicted and the actual number of tokens received to represent how accurately people can judge the trustworthiness of a novel partner after a short interaction; this served as the human baseline which will be used in section 4.3.1.

Rather than assessing purely economic decision making, interpersonal or social exchange games like the Give-Some Game have been shown by social psychologists to involve evaluations of a partner's personality, including his or her trustworthiness. For example, Fetchenhauer and Dunning (2012) had participants complete a monetary exchange game (where participants were given $5 and could gamble it for the chance to win $10). Half of the participants completed a social version of the game where they were told they were playing with another person, and their chances of interacting with a trustworthy person was 46%. The other half were told they were playing a lottery game (therefore, not involving any people) with the same probability of winning. Participants' behaviors were found to differ significantly in the social version of the game compared to the lottery version. That is, while participants made choices in the lottery that largely reflected the low probability of winning (46%), participants in the social version of the game largely ignored the given base rates (i.e., the likelihood one would encounter a trustworthy person) when making the same economic exchange decision and instead made far riskier choices. Thus, participants believed that people would behave differently than a purely probabilistic lottery, and notably, that people would be more trustworthy than stated (Fetchenhauer and Dunning, 2012). These findings suggest that behavior in social exchange games does not reflect purely economic decision making, but includes assessments or judgements of the other person involved, including their trustworthiness.

Further supporting our operationalization of trust, data from the past validation experiment in Phase 1 demonstrated that a self-report measure of how much a participant trusted a *robot partner* was significantly positively correlated with how many tokens that participant decided to give the robot [*r*(75) = 0.26, *p* < 0.05].

#### 4.1.4. Video-coded annotations

**Figure 3:**
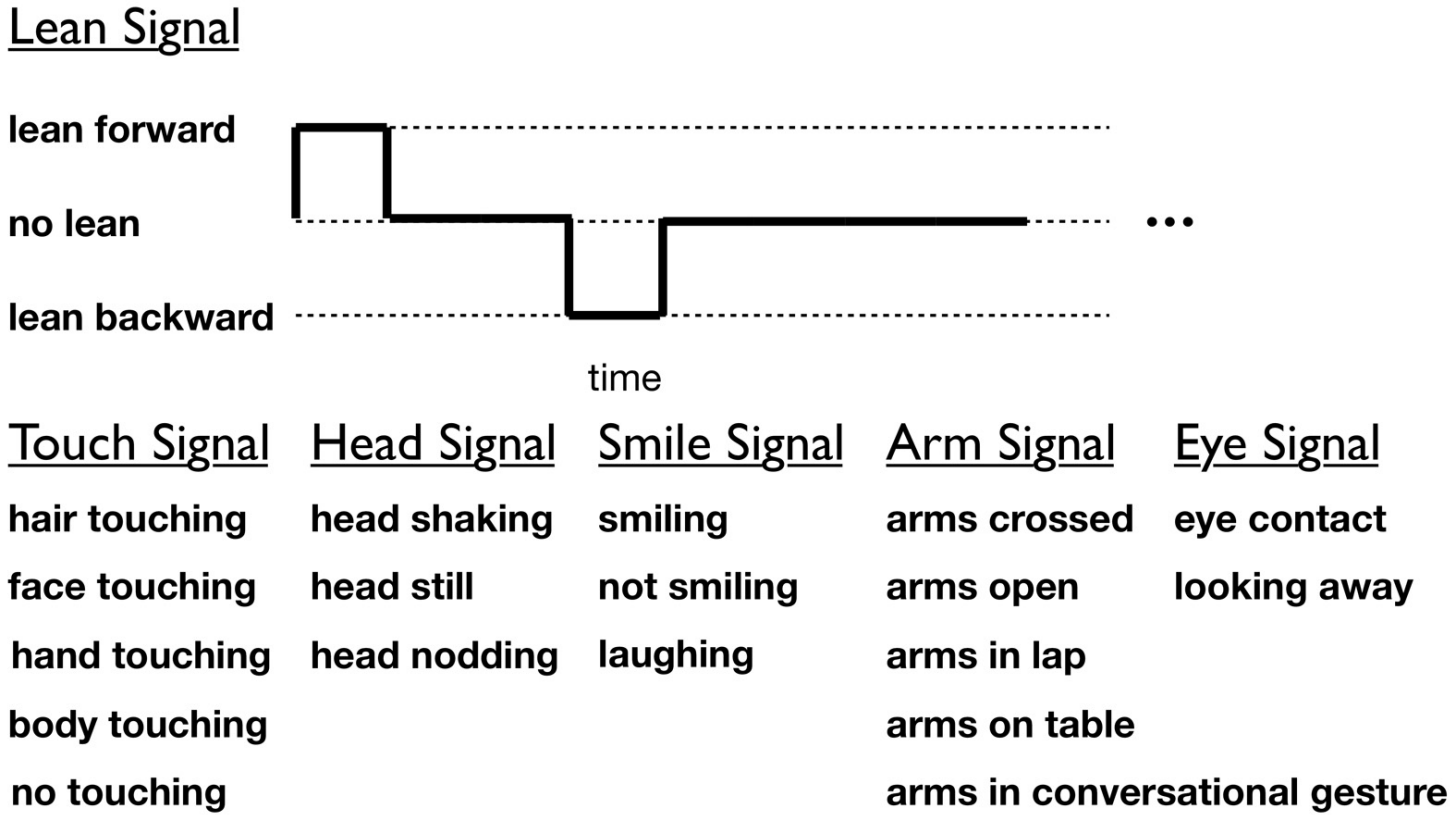
**Annotated nonverbal behaviors of participants.** Gestures within a category are mutually exclusive.

The nonverbal behaviors of the participants over the entire 5-min interaction were manually coded. The videos were coded independently by at least two coders who were blind to all hypotheses (average inter-rater reliability: ρ = 0.90). Given the high inter-rater agreement, data from only one coder is used for analysis. We coded for nonverbal behaviors that appeared frequently (by at least five participants) in the video-taped interactions. The start and stop times of the following behaviors were coded for each participant: smiling, laughing, not smiling, leaning forward, leaning backward, not leaning, eye contact, looking away, arms crossed, arms open, arms in lap, arms in conversational gesture, arms on table, hair touching, face touching, hand touching, body touching, no touching, head shaking, head nodding, and head still (see visualization and behavior categories in Figure 3). Coders were instructed to code the video for only one participant and only one nonverbal category at a time.

### 4.2. Methods for model design (phase 2)

We employ two feature engineering strategies to find a subset of features that permits effective learning and prediction. After describing our feature engineering process, we detail the training and testing procedures used for model selection and model assessment. These procedures are chosen to assess the differences in the predictive power of a model that builds a subset of features using domain knowledge as opposed to another model that narrows its selection using a popular feature-selection algorithm.

#### 4.2.1. Feature engineering

Feature engineering encompasses both extracting features that are believed to be informative and selecting an effective subset from amongst the extracted features. Feature selection is the choosing of useful features that are not redundant or irrelevant to create a predictive model with high accuracy and low risk for over-fitting (i.e., high generalizability). Domingos (2012) points out that “feature engineering is more difficult because it's domain-specific, while [machine] learners can be largely general-purpose … the most useful learners are those that facilitate incorporating knowledge.” We detail the initial full set of features that were extracted from our trust corpus, and we compare two strategies to narrow our selection of features to an effective subset. We first create a model that uses features chosen through a standard feature-selection technique called variable ranking. Leveraging our findings from Phase 1, we create another model that narrows and then extends the initial set of features by following the three guidelines listed in section 3.

***4.2.1.1 Feature extraction.*** From the video-coded nonverbal annotations, we determined how many times a participant emitted a particular cue during the interaction (e.g., 25 smile instances) and how long a participant held the cue through the duration of the interaction (e.g., for 5% of the interaction, the participant was smiling). The duration of a gesture provides additional information and has been used as a feature in other work (Bousmalis et al., 2011). For instance, if a participant crosses their arms throughout the entire interaction, then the frequency of that gesture would register as just one arms crossed. The duration measure—but not the frequency measure—reflects the prevalence of arms crossed in this scenario. The full set of features (42 in total) consists of the frequency and duration for each of the 21 nonverbal cues.

***4.2.1.2 Feature selection.*** Variable ranking is a feature-selection method commonly used in practice; this method's popularity can be attributed in part to its simplicity and scalability. Variable ranking involves independent evaluation of each candidate feature. As a consequence, it may choose redundant features and does not consider the predictive power of complementary features. Nonetheless, this method has had many reports of success in prior work (Guyon and Elisseeff, 2003). In this research, variable ranking scores each of the 42 features by the absolute value of its Pearson's correlation coefficient with respect to the number of tokens given; we then incorporate only the most highly ranked features in the trust model.

In addition to the variable-ranking model, we build a second predictive model using the domain knowledge gained from our prior work. The three guidelines derived from Phase 1 inform this model's feature selection. First, we considered the frequency and the duration of only the four trust-related cues—face touching, arms crossed, leaning backward, and hand touching—expressed by the participant (feature *x*_1_ to *x*_8_ in Table 1) and ignored other nonverbal behaviors. Secondly, since cues are more informative in their joint appearance, the model also uses as features the mean frequency and mean duration across the four trust-related cues (*x*_9_ and *x*_10_). Finally, the model draws features from the nonverbal cues not only of the participant but of the participant's partner as well. The model therefore includes the partner's gestural frequencies, durations, and mean cues (features *x*_11_ to *x*_20_); the model also includes the differences between the participant's and their partner's features to incorporate any interesting differences between their behaviors (features *x*_21_ to *x*_30_).

**Table 1:** **The 30 features for the domain-knowledge model which narrowed and extended the initial set of features by following the three guidelines from Phase 1**.

**Feature**	**Who**	**Type**	**Description**
*x*_1_ … *x*_4_	Self	Frequency	# Times gesture emitted
*x*_5_ … *x*_8_	Self	Duration	% Time gesture held
*x*_9_	Self	Joint	Mean(*x*_1_ … *x*_4_)
*x*_10_	Self	Joint	Mean(*x*_5_ … *x*_8_)
*x*_11_ … *x*_14_	Partner	Frequency	# Times gesture emitted
*x*_15_ … *x*_18_	Partner	Duration	% Time gesture held
*x*_19_	Partner	Joint	Mean(*x*_11_ … *x*_14_)
*x*_20_	Partner	Joint	Mean(*x*_15_ … *x*_18_)
*x*_21_ … *x*_24_	Diff	Frequency	*x*_*i*_ ← *x*_*i*−20_ − *x*_*i*−10_
*x*_25_ … *x*_28_	Diff	Duration	*x*_*i*_ ← *x*_i−20_ − *x*_i−10_
*x*_29_	Diff	Joint	*x*_29_ ← *x*_9_ − *x*_19_
*x*_30_	Diff	Joint	*x*_30_ ← *x*_10_ − *x*_20_

These 30 features (listed in Table 1) represent the incorporation of domain-specific knowledge in the selection process. In absence of this knowledge, the alternative method of selection is to narrow the number of features using variable ranking. The model that uses this domain-knowledge selection method will be referred to as the *domain-knowledge model*, while its naivé counterpart will be referred to as the *standard-selection model*.

#### 4.2.2. Prediction model

We aim to demonstrate the effect of incorporating domain-knowledge in the feature-selection process on the performance of a prediction model. As such, rather than exploring and comparing the predictive accuracies of various machine learning algorithms, we focus on support vector machines (SVMs) as the primary tool for our feature-focused investigation. SVMs were chosen for their wide use and prior success in modeling human behavior (Rienks et al., 2006; Kapoor et al., 2007; Jayagopi et al., 2009).

SVMs separate training examples into their classes using optimal hyperplanes with maximized margins; for a query, an SVM predicts based on which side of the class boundary the query example lies. To find effective separations, the training examples are transformed from their original finite-dimensional space into a higher dimensional feature space by a non-linear mapping function called a kernel (Hastie et al., 2009).

Each of the *m* = 112 training examples in our dataset (one per participant) contains a vector of *n* = 30 features, $\vec{x}$ = (*x*_1_, *x*_2_, …, *x*_*n*_). Features were scaled to have values within [−1, +1], preventing an over-reliance of the SVM on large-ranged features. The class label for an example is the number of tokens the participant gave their partner in the Give-Some Game, *y* ϵ {0,1,2,3,4}. With our dataset {($\vec{x}$_1_, *y*_1_), ($\vec{x}$_2_, *y*_2_), …, ($\vec{x}$_*m*_, *y*_*m*_)}, we train and test our SVM model with a Gaussian kernel using the LIBSVM library (Chang and Lin, 2011).

#### 4.2.3. Nested cross validation

To estimate the true prediction error of a model selection process, we employ a nested cross-validation method. A challenge when modeling human behavior is collecting and annotating enough real-world data to partition into three substantial training, validation, and testing sets. The training set is used to fit the models. For *model selection*, the validation set is used in tuning the model parameters to yield the lowest prediction error. For *model assessment*, the chosen model's prediction error (also called generalization error or true error) is estimated using the previously unseen testing set. In cases such as ours, when the sample size is small (*m* = 112), the method of cross validation (CV) is often used to estimate prediction error by partitioning the dataset into subsets, and in multiple rounds, each subset acts as the validation or testing set (depending on the analysis) while the remaining is used as the training set. One benefit of using cross validation is that the model can be trained from almost the whole dataset.

When using CV for both model selection and model assessment, one has to be careful that data involved in the model selection process is not reused in the final assessment of the classifier, which would occur in the case of first cross validating for model selection and then cross validating again with the same data for model assessment. When such reuse occurs, re-substitution error can falsely lower the estimate of true error (Hastie et al., 2009). We avoid such misleading results by conducting *nested* CV to obtain an almost unbiased estimate of the true error expected on an independent dataset (Varma and Simon, 2006).

We evaluate the trust models through leave-one-out nested CV and follow the nested implementation as described by Varma and Simon (2006). The leave-one-out nested CV method includes an inner loop for model selection and an outer loop for model assessment. In each iteration of the *outer loop*, a training example is removed. With the remaining examples, the best choices for hyper-parameters and features (via model selection) are determined and used to create a classifier. The resulting classifier then predicts the class of the “left out” example, resulting in some error. This process is repeated such that each training example is left out once. Prediction errors are then accumulated for a final mean prediction error (MPE) of the estimator. The MPE is calculated as the average absolute difference between the classifier's predictions and the true class labels. Of note, the nested CV process estimates the MPE of classifiers learned at every iteration of the outer loop. This provides a performance measure of an estimator (i.e., a learning algorithm) and not of a particular estimate (i.e., a single classifier).

In general, the *inner loop* performs both feature selection and hyper-parameter tuning for model selection using the remaining data from the outer loop. But in our case, for the variable-ranking selection method (described in section 4.2.1.2), a subset of features is found before the inner loop, which then conducts CV for hyper-parameter tuning. More specifically, for our SVM models, the inner loop tunes the parameters by varying the values of the model's hyper-parameters C and γ according to a grid search and chooses the values with the best prediction error, where the error is calculated by a leave-one-out CV. The cost parameter C balances the tradeoff between obtaining a low classification error on training examples and learning a large class-boundary margin, which reduces over-fitting to the training examples. In general, increasing the value of C reduces training error and risks over-fitting. The bandwidth parameter γ controls the size of the Gaussian kernel's radius, which in effect determines the smoothness of the boundary contours. Large values of γ create a less smooth boundary (i.e., higher variance), which can lead to over-fitting, whereas small values create smoother boundaries, which can lead to under-fitting. A pseudo-algorithm detailing the exact steps of our entire procedure is available in the Appendix.

Although cross-validation methods are a standard alternative for estimating prediction error when sample sizes are small, they have some limitations. In particular, leave-one-out CV can result in high variance estimates of the prediction error since the testing partition contains only one example. But in utilizing almost the whole dataset for training, the method is also regarded in achieving low biases (Japkowicz and Shah, 2011). In contrast to holdout methods (when a substantial independent test set is available), nested cross-validation does not provide an estimate of the true prediction error of a particular classifier (i.e., a single trust model) but instead reports on the average performance of classifiers built from different partitions of the data (in our case, the average performance of trust models are trained on data sets that each differ by one example).

### 4.3. Results and discussion (phase 2)

Here we discuss the prediction performance of the two computational models: the domain-knowledge model (SVM-D) and the standard-selection model (SVM-S). We then compare these models to a random model, an *a priori* model (i.e., a model that always predicts the most common class), and a human baseline.

#### 4.3.1. Results

**Figure A1:**
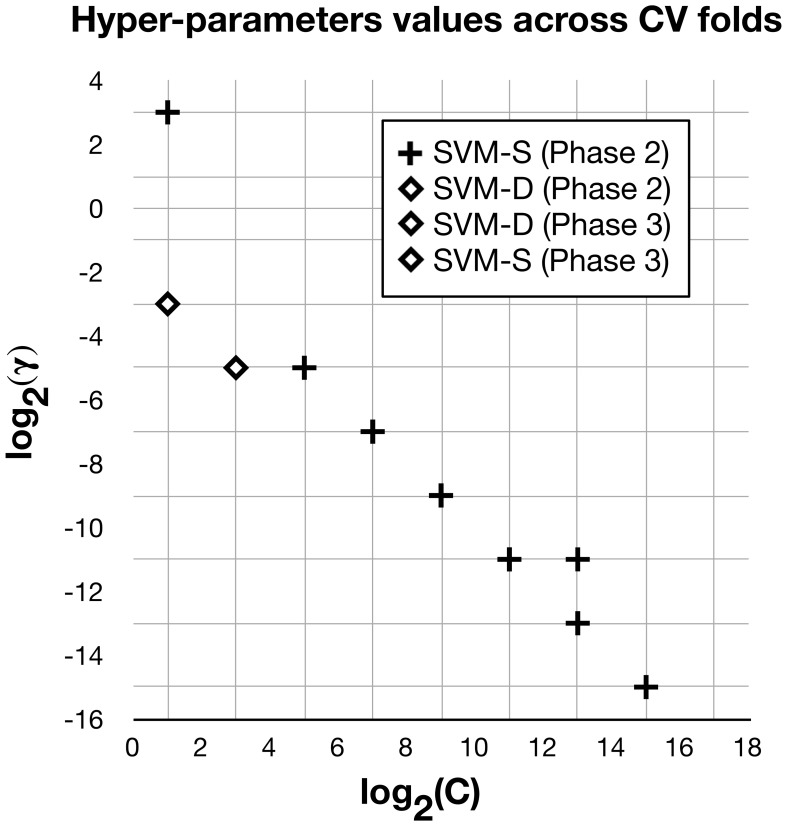
**This plot shows the hyper-parameters values C and γ selected (not showing repetitions) at different iterations of the outer cross-validation loop for the SVMs built in Phases 2 and 3.** SVM-D (in Phases 2 and 3) and SVM-S (in Phase 3) have relatively stable hyper-parameter values across cross-validation folds, while SVM-S (in Phase 2) can vary between eight sets of values.

Through leave-one-out nested CV, the SVM-D model is estimated to have a MPE of 0.74. SVM-D's hyper-parameters have values of either [C: 8, γ: 0.031] or [C: 2, γ: 0.125] at different iterations of the outer loop (i.e., across the CV folds for model assessment). The SVM-S model is estimated to have a MPE of 1.00, and its hyper-parameters vary more than the SVM-D model (see Figure A1 in the Appendix for hyper-parameter plots).

We statistically assess whether the prediction errors between classifiers are different through the Wilcoxon's Signed-Rank test (Japkowicz and Shah, 2011). Since we compare the performance of the SVM-D model to that of the SVM-S model, random model, *a priori* model, and a human baseline—resulting in four statistical tests—we counteract the increased probability of Type I error (i.e., claiming a difference where there is none) by adjusting the significance level to α = $\frac{0.05}{4}$ = 0.0125, as per the Bonferroni correction.

According to this statistical test, our SVM-D model significantly outperforms the SVM-S model (see Table 2). In comparing to other baselines, we also found the SVM-D model to significantly outperform a random model, which uniformly guesses either 0, 1, 2, 3, or 4 tokens.

**Table 2:** **The mean prediction error of the SVM-D (domain-knowledge) model, and its comparison to that of the SVM-S (standard-selection) model, *a priori* model, random model, and a human baseline**.

**Model**	**Mean prediction error**	***T*-test (α = 0.0125)**
SVM-D	0.74	–
*A priori*	0.83	*p* = 0.0173
Human	1.00	*p* = 0.0011^*^
SVM-S	1.00	*p* = 0.0004^*^
Random	1.46	*p* < 0.0001^*^

An asterisk symbol denotes statistical significance.

In Table 2, the “human” category is not actually a model but rather the participants' predictions of how many tokens their partner will give them in the Give-Some Game (as mentioned previously in section 4.1.3). These predictions served as a human baseline for how accurately people can perceive the trustworthiness of a stranger after a 5-min interaction. The SVM-D model significantly outperforms the human predictions.

**Figure 4:**
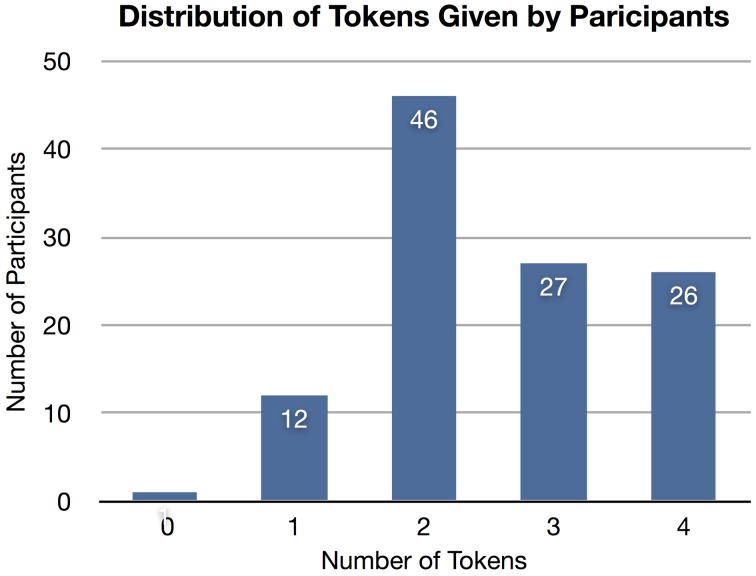
**The distribution of tokens given by participants.** The majority (41%) gave two tokens. An a priori model based on this distribution will always predict two tokens.

An *a priori* classifier ignores the nonverbal information from the features but knows the class distribution (i.e., distribution of tokens given, as shown in Figure 4). The *a priori* model always predicts the class with the lowest mean error: two tokens given. The SVM-D model outperforms the *a priori* model but not with statistical significance.

#### 4.3.2. Discussion

By incorporating the guidelines derived from Phase 1 into the feature engineering process, we designed a prediction model (SVM-D) that outperformed not only the model built in naiveté of those guidelines (SVM-S) but also outperformed human judgement.

Of note, participants gave an average of 2.58 tokens yet predicted that they would receive an average of 2.28 tokens. We believe this bias toward predicting a less generous return contributed to the error in the human predictions. Thus the SVM-D, SVM-S, and the *a priori* model all have an added advantage of being biased toward the majority class.

Our SVM-D model outperformed the *a priori* model, which ignores nonverbal behavior data. However, the difference was not significant, and so we cannot yet say with confidence that the nonverbal data improved the prediction performance of our modeling algorithm.

By identifying where our SVM-D model is making prediction errors, we can aim to find additional features that can help discriminate between the examples the model is most confused about. According to SVM-D's confusion matrix (Table 3), the model has difficulty distinguishing when an individual has a higher degree of trust toward their partner. For people who gave four tokens, the model generally predicts that two tokens will be given, contributing 55% of the total prediction error. We therefore seek to improve upon the SVM-D model in Phase 3 by deriving new features that can help differentiate individuals with greater levels of trust toward their partners, ultimately resulting in significantly more accurate predictions than the *a priori* model.

**Table 3:**
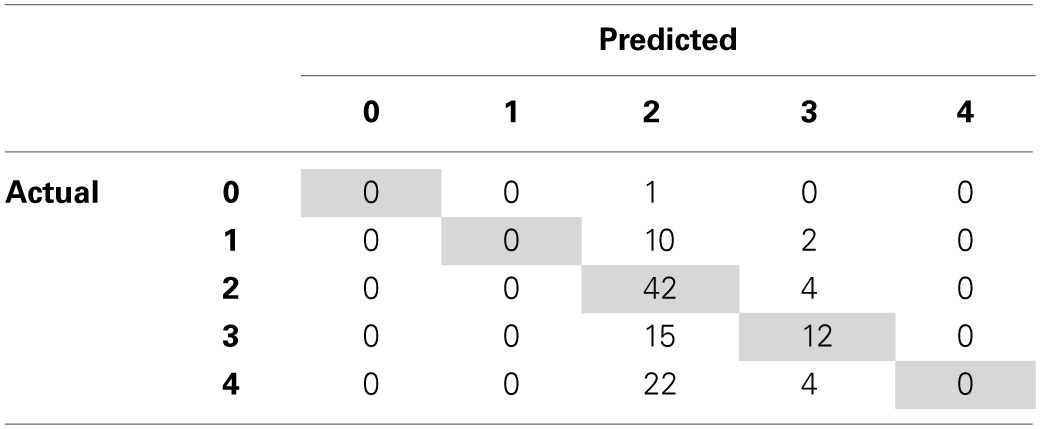
**Confusion matrix for SVM-D revealing the model having difficulty distinguishing when an individual has a higher degree of trust toward their partner**.

## 5. Temporal dynamics of trust (phase 3)

In this final phase, we improve upon the accuracy of the prediction model developed in Phase 2 by deriving additional sequence-based temporal features.

In the last part of our investigation, the model built with features chosen through domain knowledge (SVM-D) outperformed a model unaware of this knowledge (SVM-S). Additionally, SVM-D was more accurate than the *a priori* model, but not significantly so. To improve the performance of our SVM-D model, we again turn to domain knowledge to guide our search for new features.

As mentioned in Phase 1, we hypothesized that the appearance of multiple nonverbal cues together, observed in the *context* of each other, would provide reliable information about trusting outcomes. We operationalized this contextual joint appearance of the trust-related nonverbal cues through their mean frequency and mean duration as features for our trust model (described in section 4.2.1.2). These mean values attempt to roughly capture the temporal proximity of the trust-related cues occurring within a social interaction. We extend our hypothesis to another form of operationalizing “context” through the *sequence* of emitted cues. We anticipate that the contextual information given in the sequence of trust-related nonverbal cues contains predictive information not captured by the cues' mean frequencies or durations. Furthermore, rather than only observing the sequence of the four nonverbal cues associated with lower levels of trust (*low-trust cues*), we aim to observe their interplay with nonverbal cues associated with higher levels of trust (*high-trust cues*), since we anticipate more discriminating patterns to emerge from the dynamic of both high-trust and low-trust cues.

In Phase 3, we first describe the redesign of our experiment, from which we identify a set of high-trust nonverbal cues. We then present the construction of hidden Markov models (HMMs) to find temporal relationships among the new high-trust cues and the previously identified low-trust cues. From this temporal model, we derive additional features that improve upon the performance of SVM-D from Phase 2.

### 5.1. Materials (phase 3)

In this subsection, we describe the additional human-subjects experiment and analysis performed to identify a set of high-trust nonverbal cues.

#### 5.1.1. Human-subjects experiment redesign

We redesigned our previous human-subjects experiment (detailed in section 4.1) with one key difference: after the social interaction and before the game, the participants were told that the average number of tokens given is one. However, participants were also told that the average may not be indicative of what their particular partner would do and were encouraged to let their social interaction guide their predictions.

In the experiments that produced our original data (section 4.1), participants decided how many tokens to give their partner without any prior knowledge of the expected or “normal” giving behavior. These participants may have varied in their belief of how many tokens are thought to be unfairly low, fair, and over-generously high. By introducing this manipulated information about the average tokens given, we aim to shift the expectation of token-giving behavior such that individuals expect the mean level of giving to be lower (i.e., closer to one token on average compared to 2.28 tokens on average from the previous experiment).

This manipulation had two intended outcomes. First, we aim to lessen participants' variation in their beliefs of normal giving behaviors—a potential source of noise in our original data—and thereby increase the likelihood of a goodness-of-fit in how well certain nonverbal cues can predict token-giving outcomes. Second, by biasing participants toward a lower norm of one token, we aim to shift “norm-followers” away from higher levels of giving. Norm-followers are those without preference in wanting to give more or less to their partner and therefore give according to the behavioral norm. We speculate that participants that then give more than one token are most likely those trusting their partners to play cooperatively and therefore willing to deviate from the established norm. Having shifted the norm-followers away from higher levels of giving, we then expect to have more homogeneity in the variance among the nonverbal behaviors that predict higher levels of trust.

Through this manipulation, we anticipate to find a set of nonverbal cues that are significant predictors of trusting behavior in a positive direction, which we were unable to identify with the original dataset from Phase 2.

A total of 16 interaction pairs (i.e., 32 participants), again undergraduate students at Northeastern University, participated in this redesigned study (41% male and 59% female). The two independent coders of the videos for this new study were also blind to all hypotheses, including any knowledge of our previous findings. Each coder coded half of the videos from the study. To establish inter-rater reliability, each coder also coded a subset of the videos originally coded by the other independent coder (ρ = 0.93).

#### 5.1.2. Identifying high-trust nonverbal cues

To identify a set of nonverbal cues that are indicative of higher levels of trust, we employ the same procedure from our prior work. We briefly outline the procedure here (for more detail see DeSteno et al., 2012). We begin by examining the zero-order correlation between the frequency of a nonverbal cue emitted and the amount of tokens given. We identify which of the 22 nonverbal cues (i.e., those listed in section 4.1.4 with the addition of hand gesturing) positively correlate with the number of tokens given. None of the correlations were both positive and significant individually, so we again considered sets of cues. We chose candidate sets through an *ad hoc* examination of correlation coefficients and *p*-values, and we tested their joint predictive ability through a multilevel regression analysis that controls for dyadic dependencies. As we hypothesized, we were able to find a set of cues—leaning forward, smiling, arms in lap, and arms open—that positively and significantly predicted token-giving outcomes in the Give-Some Game (Φ = 0.11, *p* < 0.04).

Concluding that this cue set is indeed predictive of higher levels of trust would require experimental manipulation (as in the robotic experiment described in Phase 1) to confirm that this relationship is not merely the result of spurious correlation. But our primary interest is deriving new features that capture temporal relationships between particular cues. We therefore continue to refer the set of cues—leaning forward, smiling, arms in lap, and arms open—as indicative of higher levels of trust.

### 5.2. Methods for model design (phase 3)

Suspecting that the sequence of nonverbal cues contains predictive information not captured by the cues' mean frequencies or durations, we built a temporal model, capable of modeling processes over time. More specifically, we constructed HMMs to capture the temporal relationship between the newly identified high-trust cues and the previously identified low-trust cues using the original dataset from Phase 2 (section 4.1). Of note, the data from the redesigned experiment (section 5.1.1) is only used for the identification of the high-trust cues.

HMMs are common representations for temporal pattern recognition. However, HMMs are commonly viewed as having low interpretability; the model's internal complexity hinders any qualitative understanding of the relationship between the input and predicted outcomes (Hastie et al., 2009). Although often treated as a black box technique, HMMs are capable of finding structures that reveal interesting patterns in the data. Our technique described below demonstrates one method for leveraging an HMM's learned structure to derive new features.

#### 5.2.1. Temporal model

In applications that have a temporal progression, HMMs represent a sequence of observations in term of a network of hidden states, from which observations are probabilistically created. State *s*_*t*_ at time *t* is drawn probabilistically from a distribution conditioned on the previous state *s*_*t*−1_, and an observation *o*_*t*_ probabilistically conditioned on the current state *s*_*t*_. Thus, to model a temporal process by an HMM, three probability distributions must be learned: over initial states, *P*(*s*_0_); over state transitions, *P*(*s*_*t*_|*s*_*t*−1_); and over observations, *P*(*o*_*t*_|*s*_*t*_). In this work, the parameters for these distributions are iteratively adjusted by expectation maximization (Rabiner, 1989) to maximize the likelihood of the observation sequences in the data given the parameterized model. Possible observations were the eight high- and low-trust cues: smiling, leaning forward, leaning backward, hand touching, face touching, arms open, arms crossed, and arms in lap. Based on cues' coded start times, we extracted the sequence of only these eight gestures for each participant during their 5-min interaction. The sequence length per participant varied (min = 9, max = 87), since some individuals gesticulated these cues more often than others.

Once the initial state, state transition, and state observation probabilities are selected, a trained HMM can generate a sample sequence of observations. The simulation first generates a sample state path (i.e., Markov chain) of a given length by selecting an initial state drawn from the initial state probability distribution and selecting subsequent states based on the transition probabilities. Given a sample state path, an observation is drawn based on each of the states' observation probabilities to then form a sample sequence of observations.

When a model has many states, transition paths, and possible observations per state, deciphering the meaning of a state and its role in the state network is especially difficult. However, by simulating a trained HMM, we can qualitatively examine the generated observation sequence for informative patterns. To find discriminative patterns, we trained one HMM_low_ from participants that gave two tokens away and another HMM_high_ from participants that gave four tokens away and then searched for differences in their sample sequences of emitted nonverbal cues. Two tokens was chosen for HMM_low_ because few participants gave 0 or 1 token (leading to insufficient data for training). By comparing a sample sequence of observations from HMM_low_ and HMM_high_, we can discover any informative distinctions between their simulated outputs. For example, we could observe certain patterns of nonverbal cues that appear in succession in HMM_low_'s output that do not appear in HMM_high_'s. We can then use these unique and differentiating patterns to construct new features.

#### 5.2.2. Leave-one-out cross validation

We determined the best model parameters for HMM_low_ and HMM_high_ via leave-one-out CV. A nested method was unnecessary, since we aimed only to draw insight from the trained model and not to estimate the true prediction error.

From our original dataset from section 4.1, we have 26 training examples for HMM_high_ and 46 training examples for HMM_low_. We ran 6000 simulations using the Bayes Net Toolbox (Murphy, 2001). At every run we randomly initialized (drawn from a uniform distribution) the number of states and the initial state, transition, and observation probabilities for both of the HMMs. To determine the prediction performance of the models' parameters, we use a leave-one-out CV method, where we leave the data from one participant out and train on the remaining 71 participants for the two HMMs. The omitted example is classified as either high or low trust, determined by which of HMM_low_ or HMM_high_ has a higher log-likelihood of creating the observations in the example.

### 5.3. Results and discussion (phase 3)

Below we interpret the resulting learned structure of our HMMs and discuss the ability of the newly derived temporal features to improve on the prediction accuracy of the SVM-D model from Phase 2.

#### 5.3.1. Results

Through leave-one-out CV, our best model training result, with three states for HMM_low_ and five states for HMM_high_, has a recognition accuracy of 71% (51 hits and 21 misses) compared to 64% at chance due to the uneven distributions. Our goal, however, is not discriminative accuracy of these generative HMM models but rather to identify what interesting temporal patterns they capture. By simulating the trained HMM_high_, we get an output observation of:

*smiling → smiling → face touch → smiling → smiling → hand touch → arms in lap → arms crossed → arms in lap* …

And by simulating HMM_low_, we get an output observation of:

*smiling* → arms crossed → face touch → hand touch → smiling → face touch → arms crossed → smiling → smiling …

To make the pattern easier to decipher, we denote (+) as high-trust and (−) as low-trust cues to form:

HMM_high_ = + + − + + − + − + …

HMM_low_ = + − − − + − − + + …

Both models alternate between high-trust and low-trust cues. HMM_low_'s sequence contains frequent consecutive low-trust cues, whereas HMM_high_'s sequence contains more consecutive high-trust cues. As posited previously, these observation sequences suggest that the order of nonverbal cues may provide further information for predicting trust-related outcomes.

We use these findings to derive new features for our prediction model. Since SVMs do not naturally capture temporal dynamics, we represent the ordering of low- and high-trust gestures emitted using encoding templates. That is, by stepping through the sequence, we count the number of times (i.e., the frequency) in which we observe the following templates:

- low-trust {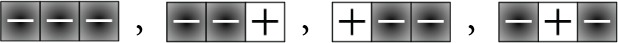}
- high-trust {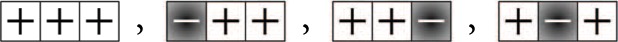}

With a sliding window of three cues, these templates in essence profile the neighboring cues (ones right before and right after a particular cue).

We considered adding the following new feature-types for our trust model: 1) high-trust templates 2) high-trust cues 3) low-trust templates. When adding the frequencies of the high-trust templates as features to our model, the MPE increased to 0.80 as compared to the previous SVM-D's MPE of 0.74 in Phase 2. When instead adding the features (that are analogous to the ones listed in Table 1) of the four high-trust nonverbal cues—leaning forward, smiling, arms in lap, and arms open—the model again increased in error with an MPE of 0.83. We therefore did not include the high-trust templates nor the four high-trust nonverbal cues into the final selection of features for our trust model.

We created 12 new features, consisting of the frequencies in which the low-trust templates are emitted by the participant, their partner, and the difference in frequency between them (shown in Table 4). Through the inclusion of the low-trust template features toward the training of a final model, our new trust model achieves an overall MPE of 0.71, which now significantly outperforms the *a priori* model (see Table 5).

**Table 4:**
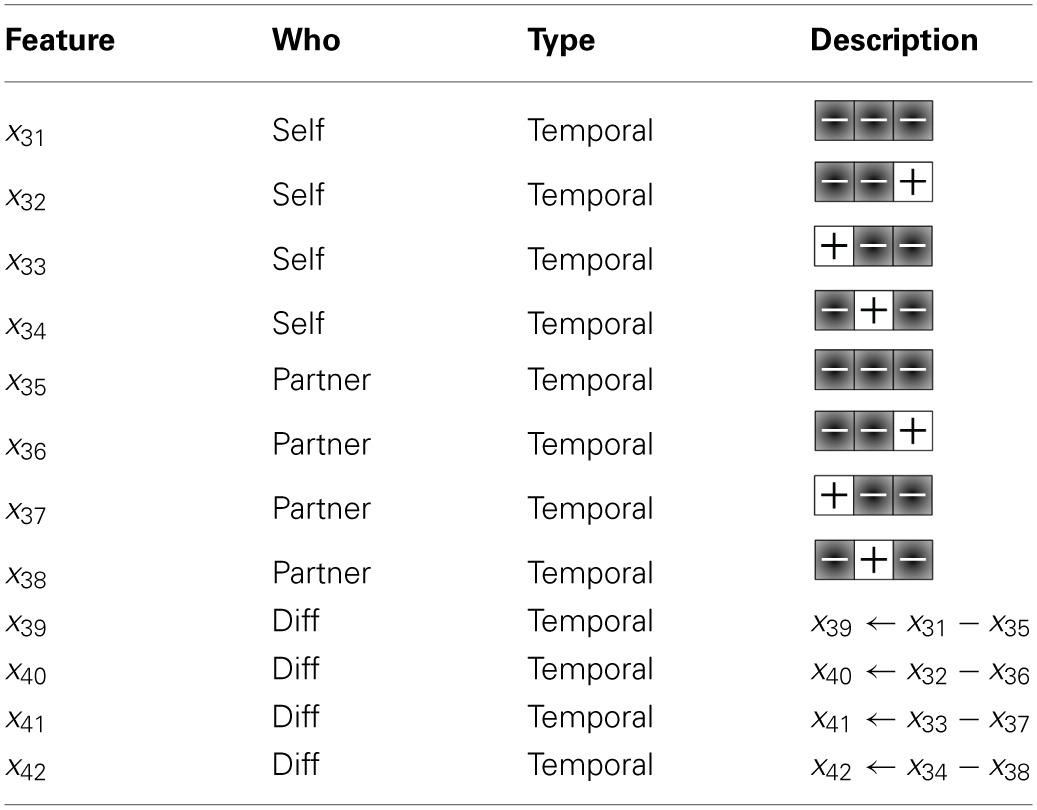
**Twelve new features, consisting of the frequencies in which the low-trust templates are emitted by the participant, their partner, and the difference in frequency between them, used to train the final SVM model**.

**Table 5:** **The updated comparisons of the baseline models to the new SVM-D model with a total of 42 features, which are listed in Tables 1, 4**.

**Model**	**Mean prediction error**	***T*-test (α = 0.0125)**
SVM-D	0.71	–
*A priori*	0.83	*p* = 0.0049^*^
SVM-S	0.86	*p* = 0.0018^*^
Human	1.00	*p* = 0.0003^*^
Random	1.46	*p* < 0.0001^*^

Of note, to maintain fair comparisons to a model not using domain knowledge, the SVM-S model uses the full 42 features detailed in section 4.2.1.1 (thus, not needing the variable ranking to find a smaller subset). An asterisk symbol denotes statistical significance.

**Figure 5:**
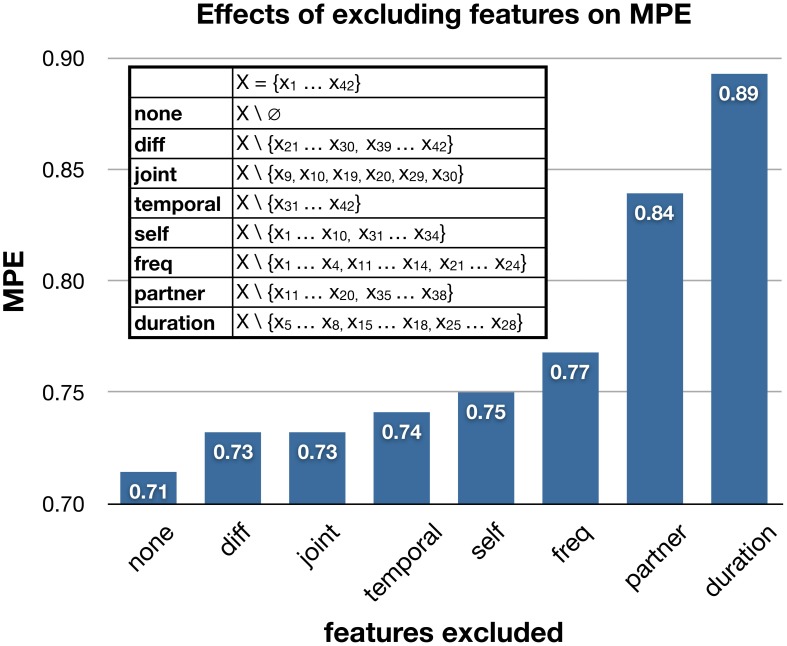
**The effects of excluding categories of features on the trust model's mean prediction error.** The legend lists the exact features of a category that were excluded, and their descriptions can be found in Tables 1, 4.

Our final trust model consists of 42 features listed in Tables 1, 4. To better understand the contribution of different components of trust signals, we performed an additional analysis to study the effects of removing particular categories of features on the trust model's performance. As shown in Figure 5, the MPE increases when excluding certain categories of features. When removing duration-type features (features *x*_5_ … *x*_8_, *x*_15_ … *x*_18_, and *x*_25_ … *x*_28_ listed in Table 1) from the full set of 42 features, the trust model's performance is most heavily effected. This suggests that the duration, or prevalence, of a gesture provides important information for the trust model. Interestingly, removing information about the partner's nonverbal behaviors has greater effects than removing the behavioral information of the individual whose trusting behavior we are trying to predict. This may suggest that when predicting the trusting behaviors of an individual, rather than directly observing their nonverbal behavior for “honest” or “leaky” signals, it is more informative to observe their partner whose behaviors greatly influence the individual's decision to trust.

#### 5.3.2. Discussion

The inability of the high-trust cues and templates to enhance the predictive power of our model is not surprising in light of the differences between the study from which the high-trust cues were identified and the original studies whose data is used to train and test our model. The experiments that provided our original data collection material (in section 4.1) were conducted in a friendly and prosocial context; as shown in Figure 4, the number of tokens participants tended to give away fell on the high or trusting end of the distribution. When the default expectation is cooperation (i.e., most participants give away high numbers of tokens), then those that deviate from this expectation are most likely the participants that did not trust their partner to play cooperatively; this scenario is the direct opposite of the one described in section 5.1.1. Thus, it is not surprising that the nonverbal cues we identified as being most predictive in these experiments were negative predictors related to lower levels of giving. In our context where the behavioral norm is for people to be more cooperative or trusting, the high-trust cues and templates lose predictive power. In line with what we observed, when adding the high-trust cues and templates as potential features, the trust model's predictive performance decreased.

### 5.4. General discussion

Our research sought to answer how a robot can (1) *behave* such that humans develop trust toward the robot (i.e., the control signal) and (2) *evaluate* the degree to which an individual trusts the robot (i.e., feedback signal). Our prior work in Phase 1 demonstrated the capacity of a social humanoid robot to influence a person's trust toward the robot through either the presence or the absence of specific nonverbal gestures. Our current work in Phases 2 and 3 demonstrates the capacity of a computational trust model to evaluate the degree to which a person trusts another social agent. An important next step is to answer how a robot can dynamically adapt its behavior to achieve or maintain a desired level of trust based on its continual reading and interpretation of a person's current level of trust toward the robot. However, before the computational trust model will readily work for a social robot in a wide range of real-world interactions, the model's current limitations will need to be addressed. We discuss three limiting dependencies of the model below.

In its current implementation, the model relies on hand-coded annotations of the nonverbal behaviors. For a robot to determine how much an individual trusts the robot, it will need to recognize these gestures autonomously. To model these gestures, 3D motion capture technology (like the Microsoft Kinect) can track the body movements of people, and gesture recognition algorithms can detect when particular nonverbal cues are being expressed. This low-level gesture recognition system can then feed into a high-level trust recognition system, which will be primarily driven by the trust model [see Lee (2011) for an initial framework].

Secondly, the trust model relies on the behavioral operationalization of trust through the Give-Some Game. A difficult but important question to consider is the game's ability to measure real-world interpersonal trust. In section 4.1.3, we provided support that behavioral trust games like the Give-Some Game do not seem to purely assess economic decision making but instead involve social evaluations of other players. We also found that subjective measures of trust (via self report) were significantly positively correlated with participants' monetary decisions in the Give-Some Game. This suggests that the Give-Some Game is capturing behavior that is related to trust behaviors in the real world, and thus we expect that the current model can generalize to predict other measures of trust or trusting behavior, particularly in situations similar to those in our experiments. However, future studies exploring how cue selection will be altered by changes in context (e.g., in situations where the default expectation is for others to be untrustworthy) will be necessary to expand the predictive ability of the current model to new contexts.

Lastly, the model relies on the contextual constraints that are implicit to the laboratory setting. The data gathered in these experiments was based on undergraduate students around the age of 18–22 attending Northeastern University in Boston, MA. The participants met unfamiliar partners (a fellow student affiliated with the same school), in a lab space (not a natural social setting), and for a short 5-min conversation. Given that the interpretation of nonverbal cues is highly context dependent, factors such as age, culture, group membership, and social environment, which are largely specified in the lab setting used in our experiments, can influence how an individual interprets trust-related social signals. The trust model is context dependent in that it has no information about these factors in its representation. Therefore, the model performs accurately when making trust judgements in the setting in which its training data originated. If we were to use this model to determine how much an interviewer trusted an interviewee, we can anticipate a drop in performance. However a model that incorporates contextual knowledge as variables can generalize to a greater variety of situations. Similarly, by incorporating other communication modalities such as facial expression, prosody, and verbal semantics, the model's predictive accuracy will most likely improve.

## 6. Conclusion

We developed a computational model capable of predicting the degree of trust a person has toward a novel partner—as operationalized by the number of tokens given in the Give-Some Game—by observing the trust-related nonverbal cues expressed in their social interaction.

Our work began with a study to demonstrate that when people have access to the nonverbal cues of their partner, they are more accurate in their assessment of their partner's trustworthiness. Confident that there is trust-related information conveyed through nonverbal cues, we furthered our investigation to identify and confirm a set of four nonverbal cues indicative of lower levels of trust and demonstrated people's readiness to interpret these same cues to infer the trustworthiness of a social humanoid robot. Through these studies, we drew three important guidelines that informed the design of our computational trust model. We demonstrated that by utilizing this domain knowledge in the feature engineering process we could design a prediction model that outperforms a baseline model built in naiveté of this knowledge.

We highlighted the importance of representing an individual's nonverbal dynamics, i.e., the relationship and influence of behaviors expressed sequentially by an individual. We represent the context within which an individual's nonverbal behaviors appear in two ways. The temporal dependency was first operationalized through the mean value of occurrence of the trust-related nonverbal cues expressed within an interaction. Then in finer temporal granularity, we investigated the sequential interplay of low-trust and high-trust nonverbal cues through the construction and simulation of hidden Markov models. Through the inclusion of new sequence-based temporal features, our computational trust model achieves a prediction performance that is significantly better than our baseline models and more accurate than human judgement.

Our multi-step research process combined the strength of experimental manipulation and machine learning to not only design a computational trust model but also to deepen our understanding about the dynamics of interpersonal trust. Through experimental design and hypothesis testing, we were able to narrow the wide field of variables to consider for our prediction model to the empirically found trust-related nonverbal cues. And by constructing a machine learning model capable of revealing temporal patterns, we discovered that the sequence of nonverbal cues a person emits provides further indications of their trust orientation toward their partner. This intersection of methodologies from social psychology and artificial intelligence research provides evidence of the usefulness of interdisciplinary techniques that push and pull each other to advance our scientific understanding of interpersonal trust.

### Conflict of interest statement

The authors declare that the research was conducted in the absence of any commercial or financial relationships that could be construed as a potential conflict of interest.

## Figures and Tables

**Algorithm 1:**
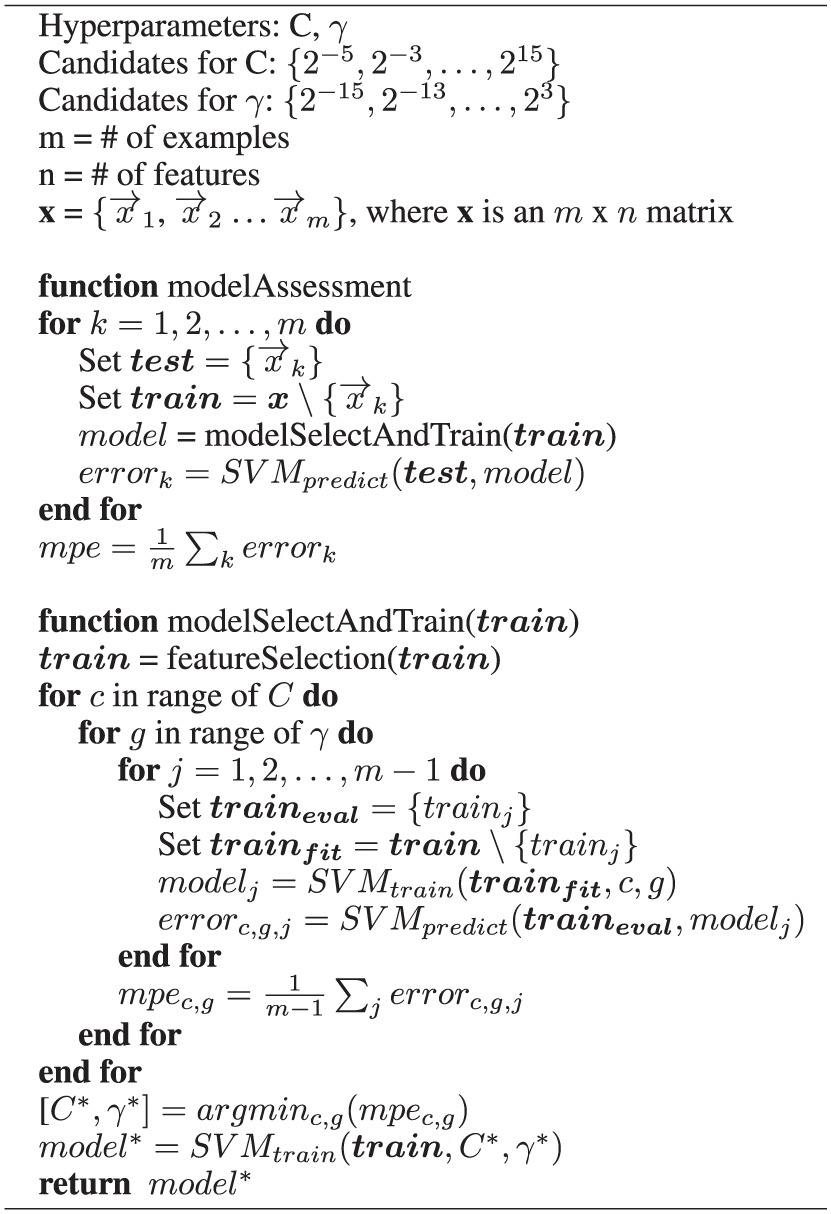
Leave-one-out Nested Cross Validation
